# A Compact Multiple Notched Ultra-Wide Band Antenna with an Analysis of the CSRR-TO-CSRR Coupling for Portable UWB Applications

**DOI:** 10.3390/s17102174

**Published:** 2017-09-25

**Authors:** MuhibUr Rahman, Dong-Sik Ko, Jung-Dong Park

**Affiliations:** 1 Division of Electronics and Electrical Engineering, Dongguk University, Seoul 04620, Korea; muhib@dongguk.edu; 2 Poongsan Corporation Ltd., Daejeon 34027, Korea; abito99@poongsan.co.kr

**Keywords:** UWB antenna, multiple notches, complementary split ring resonator (CSRR), UWB radar

## Abstract

We present a compact ultra-wideband (UWB) antenna integrated with sharp notches with a detailed analysis of the mutual coupling of the multiple notch resonators. By utilizing complementary split ring resonators (CSRR) on the radiating semi-circular patch, we achieve the sharp notch-filtering of various bands within the UWB band without increasing the antenna size. The notched frequency bands include WiMAX, INSAT, and lower and upper WLAN. In order to estimate the frequency shifts of the notch due to the coupling of the nearby CSRRs, an analysis of the coupling among the multiple notch resonators is carried out and we construct the lumped-circuit equivalent model. The time domain analysis of the proposed antenna is performed to show its validity on the UWB application. The measured frequency response of the input port corresponds quite well with the calculations and simulations. The radiation pattern of the implemented quad-notched UWB antenna is nearly omnidirectional in the passband.

## 1. Introduction

Ultra-wideband (UWB) antennas have widely drawn considerable attention since the Federal Communication Commission (FCC) in the USA assigned the frequency range from 3.1 to 10.6 GHz to UWB wireless indoor communication applications. There are several advantages of UWB systems for communications and sensing applications. UWB communications take advantage of low-power consumption, high data rate, robustness to the multipath environment, relatively low complexity and high time-domain resolution [1]. In sensing applications, UWB radar has been in the spotlight recently for various medical applications [2], microwave imaging sensor [3], indoor human detection and motion sensing [4,5,6], owing to its high precision ranging and its robustness in multipath as well as relatively low-power consumption. In UWB system design, compact and cost-effective UWB antennae are critical for a high-performance system, especially in portable applications. In [7], state of the art geometry, manufacturing technologies, materials, and numerical techniques, adopted for the analysis and design of a various class of UWB antennas for sensing and communications, are reported. 

Several narrow-band systems exist within the UWB frequency band, such as wireless local area network (WLAN), worldwide interoperability for microwave access (WiMAX), and the Indian national satellite (INSAT) frequency bands operating at 3.30–3.60 GHz, 5.15–5.825 GHz and 4.50–4.80 GHz, respectively. The WLAN frequency band is further divided into two narrow frequency bands including lower WLAN at 5.15–5.35 GHz and upper WLAN at 5.70–5.825 GHz. Therefore, these narrow-band signals, with relatively high power in the UWB, can seriously contaminate the UWB spectrum without proper narrow-band signal rejection schemes.

Several methods have been proposed to achieve efficient narrow-band rejection in the literature. In [8,9,10], they introduced etching slots on the radiating elements to obtain notched bands in UWB antennas. However, the proposed antennas with etching slots have the undesirable wideband rejection characteristics from 5 GHz to 6 GHz other than the desirable narrowband notch at 5.15–5.35 GHz and 5.70–5.825 GHz for lower WLAN and upper WLAN bands, respectively. Due to this reason, the UWB system may lose any essential data, which results in a bad quality of the received signals and degradation of the received signal within the range of 5.35–5.70 GHz. Similarly, in [11] three resonating elements were constructed on top of the modified ground plane to achieve tri-band notching and reduce coupling between the resonators. Correspondingly, in [12] they introduced a triple band-notched UWB antenna that utilizes three miniaturized electrical resonators, being considered as a capacitively loaded loop element. An UWB quad band-notched antenna has been proposed in [13], which can filter the WiMAX, INSAT, lower WLAN and upper WLAN bands. However, the proposed antenna is relatively bulky in size (33.5 × 30 × 2.34 mm^3^) and complex to implement since the antenna has been designed on four multilayered surfaces to reduce the coupling of each resonator. Moreover, the implemented notch filters in [12,13] are not sharp enough, and their frequency edge selectivity is relatively weak. Therefore, it is quite a challenging task to implement an efficient notching technique with compact size due to the strong coupling between each notching element when each resonant frequency is close to each other (e.g., lower and upper WLAN bands). To achieve a compact UWB antenna with multiple band rejection characteristics, we need to investigate the effect of coupling caused by each resonator to determine the notching frequency accurately.

In this paper, we present an ultra-wideband (UWB) antenna integrated with CSRR notching resonators at WiMAX, INSAT, and lower and upper WLAN bands, each of which is placed nearby that has strong coupling among them. We present a detailed analysis of a coupling coefficient matrix of the notches. We describe design and simulations of an UWB antenna with quad-notches by introducing four CSRRs on the conventional radiating semi-circular patch in Section 2. To estimate the notch frequencies from the multiple CSRRs caused by the coupling of each resonator, the mutual inductance of each slot resonator is calculated from the equivalent SRR, which is the silhouette of CSRR based on the Babinet’s principle, and the resonators effect on each other is taken into account. We also present the frequency response of the antenna input port with the effect of mutual inductance caused by each resonator on the others. Based on this analysis, the corresponding equivalent lumped element circuit is developed and analyzed in Section 3. The time domain resolution of the reference and proposed antenna is also discussed in Section 4, with the response of group delay and received waveform at a different distance. Implementation and measurement results are presented in Section 5, while the comparison of the proposed antenna with the reported state of the art designs is discussed in Section 6, then followed by the conclusion in Section 7. 

## 2. Design and Simulation Results

The multiple-notched UWB antenna is developed by implementing CSRRs on top of the reference UWB antenna. The reference UWB antenna is symmetrically arranged at its center line and consists of a semi-circle shaped radiator and partial ground plane. As the center frequency of the UWB spectrum is 5.50 GHz, the radius of the designed patch was 15.5 mm calculated by following Equation [14]:

(1) $$R=\frac{K}{\sqrt{1+\frac{2h}{\pi \varepsilon_{eff}K\left(\ln\frac{\pi K}{2h}+1.7726\right)}}},\;K=\frac{8.791\times 10^{9}}{f_{r}\sqrt{\varepsilon_{eff}}},$$

where *f_r_* is the resonance frequency, *ε_eff_* is the effective dielectric constant, *R* is the radius of the patch and *h* is the substrate height. The radiating patch was implemented on a 20 mil Rogers RO4003 substrate with a dielectric constant of 3.55 with a loss tangent of 0.0027.

**Figure 1:**
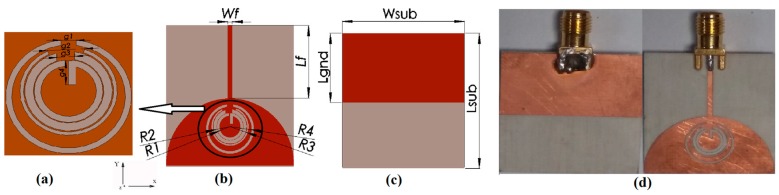
Geometry of the proposed antenna, (**a**) Magnified view of the CSRR, (**b**) Front View, (**c**) Rear-View, and (**d**) Realized prototype.

The reference UWB antenna is further modified to reject the interfering bands that fall within the UWB frequency band. To obtain the notching response, we inserted four resonators implemented with CSRRs on the radiating patch, where each CSRR is producing single notch band. Around the designed notch-frequencies, the radiation is obstructed, and antenna input impedance becomes drastically different from that in the passband. The optimal position of the CSRRs is judged based on the surface current distribution analysis. At notch frequencies, the current flows are more dominating around the resonant structures. Dimensions of the proposed antenna are presented in Table 1 and illustrated in Figure 1. The stopband frequency corresponding to upper WLAN, lower WLAN, INSAT and WiMAX bands are approximately calculated by [13]:

(2) $$f_{i}=\frac{c}{2\pi r_{i}\sqrt{\frac{\varepsilon_{r}+1}{2}}},$$

where *ε_r_* is the relative permittivity of the substrate, *r_i_* is the radius of the *i*th ring, *c* is the speed of light, and *f_i_* is the resonant frequency of the corresponding notch with *i* = 1, 2, 3, and 4.

### 2.1. VSWR (Reflection Coefficient) and Antenna Gain (Radiation Efficiency)

**Figure 2:**
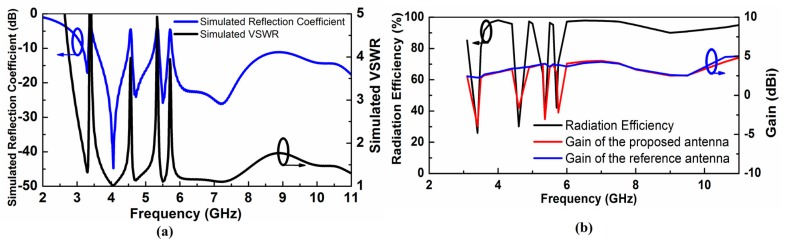
(**a**) Simulated Voltage Standing Wave Ratio (VSWR) and antenna reflection coefficient (**b**) Radiation efficiency and antenna gain versus frequency.

We perform simulations of the proposed antenna with CST Microwave Studio Suite^TM^. The designed antenna functions properly over the complete UWB frequency band while it effectively rejects the WiMAX (3.30–3.60 GHz), INSAT (4.5–4.7 GHz), lower WLAN (5.15–5.35 GHz) and upper WLAN (5.72–5.8 GHz) frequency bands. Figure 2a shows the simulated VSWR and reflection coefficient of the proposed quad-notched UWB antenna. Figure 2b presents the UWB antenna gain with and without stop bands. The antenna gain and radiation efficiency are suppressed well at the notched frequency bands owing to the increased VSWR obtained from the multiple numbers of CSRRs.

### 2.2. Notched Frequency Control

**Figure 3:**
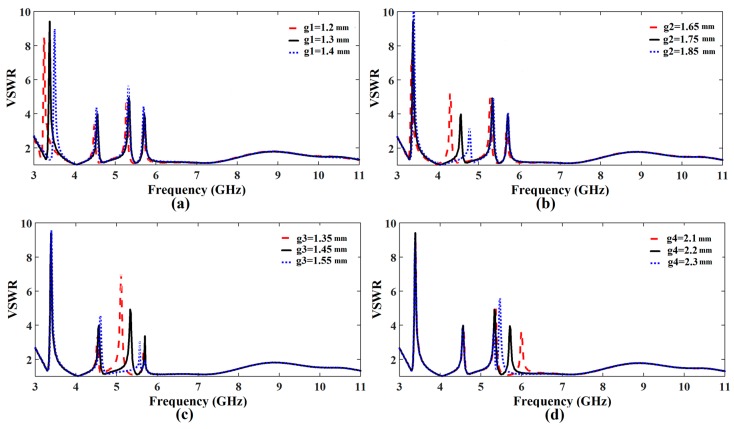
Notched frequency control (**a**) Adjusting notch at WiMAX frequency obtained by varying the geometrical parameter g1 (**b**) Adjusting notch at INSAT frequency obtained by varying the geometrical parameter g2 (**c**) Adjusting notch at lower WLAN frequency obtained by varying the geometrical parameter g3 (**d**) Adjusting notch at upper WLAN frequency obtained by varying the geometrical parameter g4.

We can easily control each notching frequency by tuning design parameters presented in Figure 1. The WiMAX, INSAT and lower WLAN band are adequately tunable by increasing or decreasing g1, g2, and g3, which are the gaps between the first, second and third outer rings, respectively as shown in Figure 1a. Figure 3a–c present the tuning characteristics of these filtering frequency bands for WiMAX, INSAT and lower WLAN frequency bands, respectively.

To achieve an accurate notching frequency of the WLAN band, we combined the corresponding CSRR with a rectangular slot resonator. Since the rectangular slot resonator can be easily implementable without area consumption, we can easily increase or decrease its length, which shifts the notching frequency to higher and lower, respectively. This behavior of adjusting the upper WLAN frequency band is shown in Figure 3d where the length of the rectangular slot resonator g4 is used to tune the notching frequency.

## 3. Analysis of Quad-Notched Antenna with CSRR-TO-CSRR Coupling

The SRR and CSRR being dual of each other have caught lots of attention in antenna designing and filtering of interfering bands in UWB frequency range [15,16]. Although the SRR and CSRR technique is very effective in providing notching response, applying this technique for closely spaced notching frequencies has been quite limited due to the mutual coupling between each element. The easiest approach to solving the coupling issue is to separate away from each resonator physically so that the effect of coupling is negligible each other. However, this method makes the size of the antenna quite bulky. Instead, we predict the frequency shift of the relatively highly coupled resonators, and it can achieve desirable filtering behavior with least amount of iterations at the design stage.

### 3.1. Calculation of the Coupling Coefficients

To calculate the coupling among CSRRs, we have analyzed its silhouette, SRR rings implemented based on the Babinet principle [17]. The Babinet principle provides the conversion relation between the input impedance of the radiating metal and its silhouette slot using the following relation:

(3) $$Z_{metal}.Z_{slot}=\frac{\eta^{2}}{4},$$

where η is the intrinsic impedance of the medium in which the assembly is embedded, *Z_metal_* and *Z_slot_* are input impedances of the radiating metal, and its counterpart slot, respectively.

The SRR creates the same coupling as its dual counterpart in that scenario. Each SRR is excited separately, and we obtain the corresponding scattering matrix (S-matrix) in the 3D EM simulator. After the extraction of the scattering matrix, we have calculated the impedance matrix (Z-matrix) from the corresponding scattering matrix. The inductance matrix (M-matrix) is then extracted from the Z-matrix using Equation (4). Finally, the coupling coefficient matrix (K-matrix) is derived from the M-matrix from Equation (4). The generalized approach of the coefficient calculation is shown in Equation (4), while the specific matrix for the case of four SRR elements is shown in Equation (5). The SRR elements decide the order of the matrix to be implemented for a particular case. The corresponding detailed conversion sequence is described in Equation (5), and the matrix extraction is performed at a frequency much lower than the lowest resonance frequency of the notches.

(4) $$S_{ij}\to Z_{ij}\to M_{ij}=\frac{img(Z_{ij})}{2\pi f}\to K_{ij}=\frac{M_{ij}}{\sqrt{M_{ii}M_{jj}}},$$

(5) $$\begin{array}{l} \;{\left[\begin{array}{cccc} S_{11} & S_{12} & S_{13} & S_{14}\\ S_{21} & S_{22} & S_{23} & S_{24}\\ S_{31} & S_{32} & S_{33} & S_{34}\\ S_{41} & S_{42} & S_{43} & S_{44} \end{array}\right]}_{S(4\times 4)}\;\to \;{\left[\begin{array}{cccc} Z_{11} & Z_{12} & Z_{13} & Z_{14}\\ z_{21} & Z_{22} & Z_{23} & Z_{24}\\ Z_{31} & Z_{32} & Z_{33} & Z_{34}\\ Z_{41} & Z_{42} & Z_{43} & Z_{44} \end{array}\right]}_{Z(4\times 4)}\\ \;↓\\ {\left[\begin{array}{cccc} K_{11} & K_{12} & K_{13} & K_{14}\\ K_{21} & K_{22} & K_{23} & K_{24}\\ K_{31} & K_{32} & K_{33} & K_{34}\\ K_{41} & K_{42} & K_{43} & K_{44} \end{array}\right]}_{K(4\times 4)}\leftarrow \;{\left[\begin{array}{cccc} M_{11} & M_{12} & M_{13} & M_{14}\\ M_{21} & M_{22} & M_{23} & M_{24}\\ M_{31} & M_{32} & M_{33} & M_{34}\\ M_{41} & M_{42} & M_{43} & M_{44} \end{array}\right]}_{M(4\times 4)} \end{array},$$

**Figure 4:**
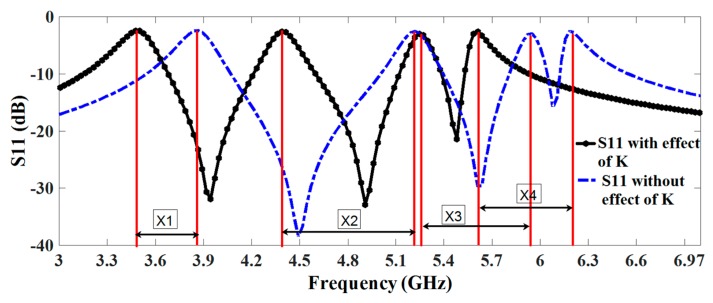
Corresponding shift in the frequency response of each rejection band caused by coupling effect.

After applying the above-generalized technique, the effect of antenna response is observed with and without coupling. It shows that there exists a significant amount of coupling among the CSRR elements and the corresponding shift in the frequency is presented in Table 2 as well as Figure 4. The INSAT and lower WLAN resonator elements are placed in the middle where we can observe a strong coupling between these two resonators. With the help of the proposed analysis, the resonance frequency of each resonator can be more easily adjustable, by taking into account the effect of coupling. By introducing the effect of this coupling in the lumped element model, we will accurately judge the corresponding frequency shift that has been caused by the mutual coupling of the nearby resonator. 

### 3.2. Equivalent Lumped Element Circuit

**Figure 5:**
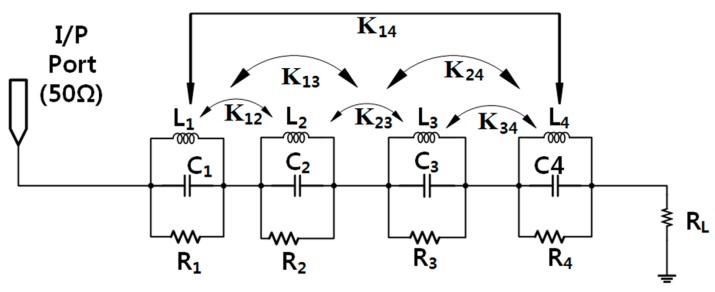
Equivalent lumped-element circuit model of the corresponding CSRR.

**Figure 6:**
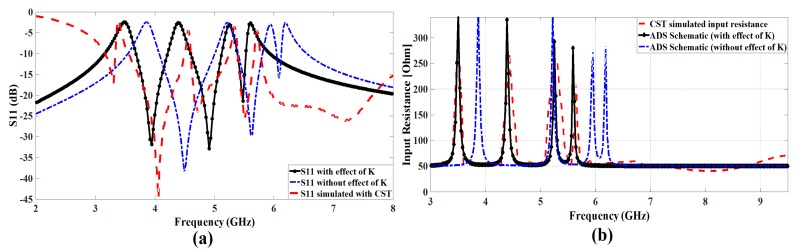
(**a**) Magnitude of the S11 parameter (**a**), and (**b**) of the antenna input resistance versus frequency. Results computed using Computer Simulation Technology (CST) and Advanced Design System (ADS) software tools.

We model the presented antenna impedance as a lumped-element circuit with four parallel RLC resonators connected in series for the four notched bands and a radiation resistance of the reference antenna as a load as shown in Figure 5. The first parallel RLC resonator resonates at 3.5 GHz, the second at 4.6 GHz, the third at 5.24 GHz and the fourth at 5.78 GHz while the load is the radiation resistance of the antenna. The input resistance of the circuit becomes maximum at the notched frequency, which validates the filtering effect as shown in Figure 6b.

The quality factor (*Q*_0_) of the RLC resonators of lumped elements at notched bands clarify the sharpness of the filtering. The frequency behavior of the antenna input impedance can also be observed from the lumped element equivalent model where the values of the lumped elements utilized in the equivalent circuit are given by [18]:

(6) $$Q_{0}=\frac{f}{BW},$$

(7) $$Q_{0}=2\pi f_{0}RC,$$

(8) $$f_{0}=\frac{1}{2\pi \sqrt{LC}},$$

where *f*_0_ is the resonant notched frequency, *C* is the capacitor, *R* is the resistor, and *L* is the inductor of RLC circuit. The values of the resistors obtained from the 3-D EM simulation are 310 Ω for the first RLC circuit, 295 Ω for the second RLC circuit, 240 Ω for the third RLC circuit, and 270 Ω for the fourth RLC circuit. After that, first *Q*_0_ is calculated and then lumped element values are determined at 3.50 GHz, 4.60 GHz, 5.24 GHz, and 5.78 GHz. Then, the effect of coupling among the notching resonators is considered in the form of the M-matrix, which are extracted from the equivalent SRR’s. Table 3 lists the values of the lumped elements extracted using Equations (6)–(8) for the design consideration of the equivalent circuit. Thus, the equivalent circuit is first simulated without coupling effect, and it is simulated by introducing coupling effect and both the response is correlated in Figure 4. We can see the effect of the coupling, and the equivalent lumped-element circuit model simulated in ADS (Advanced Design system of Keysight Technologies) corresponds well with the response taken from CST^TM^ as presented in Figure 6.

## 4. Time Domain Behavior of the Proposed Antenna

**Figure 7:**
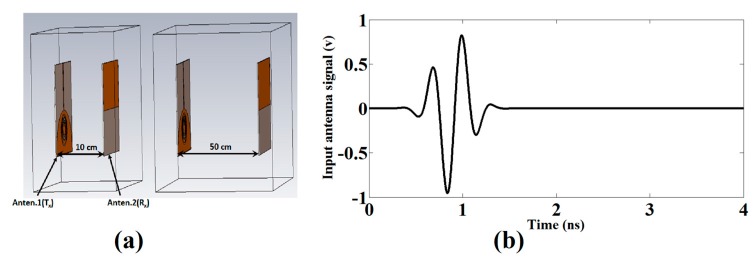
(**a**) Setup of the proposed antenna at two different distance (**b**) Input pulse selected for the excitation of the proposed antenna.

Figure 7a shows the setup that uses the two identical antennas, one acting as a transmitter (T_x_) and another as a receiver (R_x_) where the transmitting antenna is fixed while the receiving antenna has been moved to 10 cm, 30 cm and 50 cm. The antennas are excited using the 5th order Gaussian pulse which essentially satisfies the FCC spectral mask for UWB communications [19]. The applied 5th derivative of the Gaussian pulse is given by:

(9) $$s_{1}(t)=GM_{5}(t)=C\left(-\frac{t^{5}}{\sqrt{2\pi }\sigma^{11}}+\frac{10t^{3}}{\sqrt{2\pi }\sigma^{9}}-\frac{15t}{\sqrt{2\pi }\sigma^{7}}\right)\times \exp\left(\frac{t^{2}}{2\sigma^{2}}\right),$$

where *C* must be chosen to fulfill the FCC obligations of peak power spectral density equal to 51 ps, which ensures that the profile of the spectrum obeys within FCC limits. Figure 7b shows the single pulse used for excitation.

We have observed the response of the antenna with a front-to-front scenario at 10 cm, 30 cm and 50 cm. The receiving and transmitting antenna signal is provided and correlated in each case. To calculate the correlation between the transmitted input pulse and received pulse, the following equation is utilized to find out the correlation coefficient [20].

(10) $$\rho =\underset{\tau }{\max}\left[\frac{\int s_{1}(t)s_{2}(t-\tau )dt}{\sqrt{\int s_{1}{}^{2}(t)dt}\sqrt{\int s_{2}{}^{2}(t)dt}}\right],$$

where τ represents the delay and ρ is the correlation coefficient whose values at different receiving distances are summarized in Table 4.

The stretch ratio of the pulse width is also examined, which is an important figure-of-merits for UWB pulse radio. Since we know that energy is concentrated mostly around the peak and the time window comprising a certain percentage of the entire energy is termed as the pulse width [21], the ratio of the width of the far-field signal waveform intensity to the source signal is defined as the stretch ratio of the pulse width. The stretch ratio (SR) that captures the energy up to 90% is given by:

(11) $$SR=\frac{E_{sr}{}^{-1}(0.90)-E_{sr}{}^{-1}(0.10)}{E_{st}{}^{-1}(0.90)-E_{st}{}^{-1}(0.10)},$$

where $E_{sr}$ and $E_{st}$ are the corresponding normalized received, and the transmitted cumulative energy function and can be given as:

(12) $$E_{s}(t)=\frac{\int_{-\infty }^{t}{|s(t)|}^{2}dt}{\int_{-\infty }^{\infty }{|s(t)|}^{2}dt},$$

**Figure 8:**
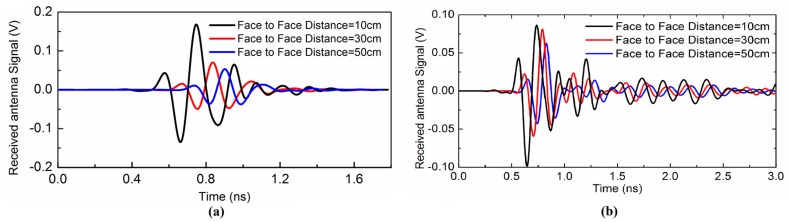
(**a**) Simulated received waveform of the reference antenna placed at three different positions (**b**) Simulated received waveform of the proposed antenna placed at three different positions.

**Figure 9:**
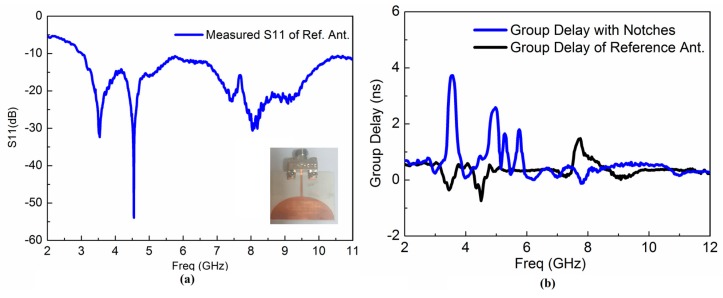
(**a**) Measured S11 response of the reference UWB antenna (**b**) Group Delay of the reference vs. proposed multiple notched UWB antenna.

The group delays and received waveform response of the proposed antenna are calculated to examine the time domain characteristics. Figure 8a,b present the received waveform of the reference and proposed antenna, respectively. The achieved results demonstrate that the proposed antenna has good linear transmission performance and could be useful for UWB radio applications as well as breast cancer detection applications. We implemented and measured the corresponding reference UWB antenna to evaluate the operation of the proposed antenna in the UWB range as shown in Figure 9a. Figure 9b presents the measured group delay response of the reference and proposed UWB antenna. In order to check the validity of the proposed antenna in the time domain, the reference antenna is designed and fabricated, and its corresponding group delay is correlated with the proposed antenna. The measured reference antenna response is shown in Figure 9a and clarifies that the reference antenna operates in the overall UWB frequency band. Additionally, it can be used as a template antenna. The proposed antenna in reference with the template UWB antenna displays a stable group delay in UWB frequency band having variation less than 1 ns in the overall UWB frequency band except in the notched bands centered on 3.5, 4.6, 5.25 and 5.8 GHz where the group delay variation becomes large. The group delay variation extends from 2 ns to 4 ns at the notched frequency bands. 

## 5. Measurements

We fabricated the compact quad-notched UWB antenna as shown in Figure 1d. A corresponding reference UWB antenna has also been implemented and measured as illustrated in Figure 9a. The group delay response of the reference and proposed UWB antenna were measured to investigate time domain behavior of the implemented antenna, as shown in Figure 9b. 

In the overall UWB frequency band, the proposed antenna, compared with the reference UWB antenna, displays a stable group delay in the UWB frequency band having variation less than 1 ns except the notched bands centered at 3.5, 4.6, 5.25 and 5.8 GHz where the group delay variation became significant. The group delay variation increases from 2 ns to 4 ns at the notched frequency bands.

**Figure 10:**
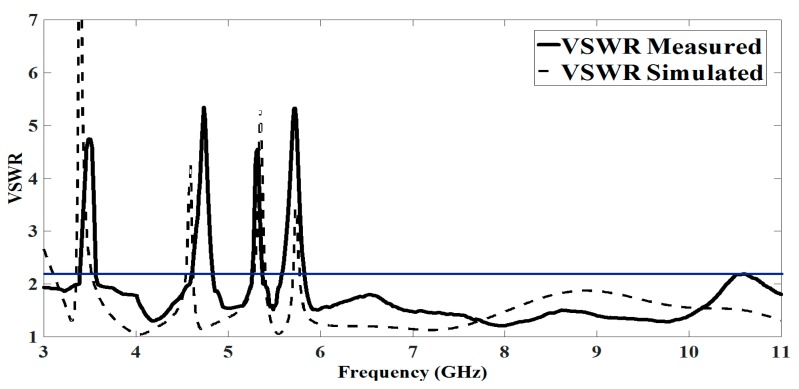
Simulated vs. measured VSWR of the proposed antenna.

A good agreement is observed between the simulated and measured results of the multiple-notched UWB antenna for the filtering behavior as shown in Figure 10. The slight discrepancy between the simulated and measured results is due to the mismatch from the SMA connector that has not been considered in the simulation.

**Figure 11:**
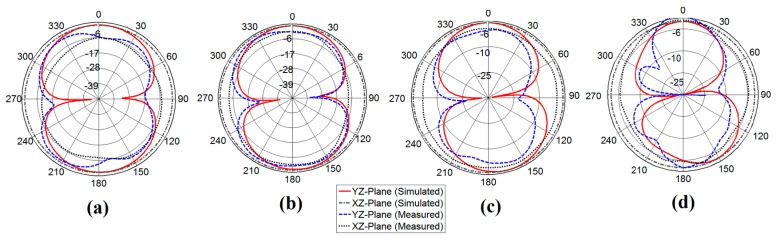
Simulated vs. measured radiation pattern of the proposed antenna (**a**) 3.8 GHz (**b**) 4.9 GHz (**c**) 6.24 GHz (**d**) 8.15 GHz.

The radiation pattern measurements in the XZ-plane and YZ-plane were carried out using anechoic chamber. The chamber is equipped with the near-field planner scanner and a far-field tower to test and measure the radiation pattern of the antenna under test (AUT). We measured the co-polarized beam patterns at 3.8, 4.9, 6.24 and 8.1 GHz which cover most of the wireless interferences at lower, upper, and mid frequencies of the UWB and the measured radiation patterns show nearly omnidirectional as expected as presented in Figure 11. The uniformity is also evaluated to assess the radiation pattern quantitatively, which is defined as the number of measured points in the radiation pattern whose deviation from the peak value is less than 6 dB divided by the total number of measured points in a single plane cut [22].

The radiation pattern is measured in an anechoic chamber from 3 to 11 GHz with a 0.0249 GHz step interval. The corresponding frequency points from the step interval is calculated as 401, and the co-polarized radiation pattern satisfies the condition as below:

(13) $$Uniformity=P\left[{|S_{21}{,}_{AUT}(\theta_{\max},\phi_{\max})|}_{\left(dB\right)}-{|S_{21,AUT}(\theta ,\phi )|}_{\left(dB\right)}\leq 6dB\right],$$

where *S*_21*,AUT*_ *(θ*, *ϕ)* is the measured radiation pattern of the antenna under test at a particular plane cut at given frequency, and *(θ_max_*, *ϕ_max_)* is the particular cut where the maximum radiation pattern is observed. The extracted uniformity from measured radiation pattern data at 3.8, 4.9, 6.24 and 8.15 GHz are calculated as 0.975, 0.933, 0.845 and 0.705, respectively. The extracted uniformity of the antenna remains larger than 0.8 for frequencies up to 6.5 GHz and finally degrades at higher frequencies due to unwanted higher order modes.

## 6. Comparison with Recently Proposed Designs

We compared the proposed UWB antenna with other recently reported UWB antennas in the literature. Table 5 reveals that there is a distinct trade-off between the antenna size and the performance of filtering of the UWB antenna, owing to the coupling effect. Moreover, miniaturization also causes a degraded radiation pattern as well as low radiation efficiency. With this regard, the proposed technique has a distinct advantage over other state of the art designs in the context of the increased number of the multiple notches with compact size as this technique consumes less space owing to the careful analysis of their mutual coupling effect. The proposed antenna also exhibits a stable radiation pattern and wide bandwidth over the whole range of UWB passband.

## 7. Conclusions

A compact UWB antenna having quad-notched bands was presented. The quad-notched bands were obtained by integrating four compact CSRRs on a semi-circular radiating patch. The antenna was designed to operate as an RF sensor or to be installed in portable UWB devices. The corresponding mutual couplings among each CSRR were analyzed with the help of the Babinet principle. With this approach, the frequency shift of the notches caused by the strong coupling among the CSRRs was evaluated. Moreover, an equivalent circuit of the proposed antenna was derived, while a UWB time domain analysis was performed to check its validity in practical applications. The measurements carried out on an antenna prototype match well with the numerical simulations. In conclusion, the proposed antenna is widely applicable in the field of UWB communications and sensing applications owing to its compactness with multi-band notches.

## Figures and Tables

**Table 1:** Dimensions of the proposed quad-notched ultra-wide band (UWB) antenna.

Parameter	Value (mm)	Parameter	Value (mm)
Lsub	30	Lgnd	15.3
Wsub	28	Lf	15.5
Wf	1	R1	2.3
R2	2.5	R3	3.8
R4	5.2	g1	1.3
g2	1.75	g3	1.45
g4	2.2		

**Table 2:** Effect of coupling matrix on the resonance of notched bands.

	*f_(WiMAX)_*	*f_(INSAT)_*	*f_(lower WLAN)_*	*f_(Upper WLAN)_*
Predicted without coupling (*f*_1_)	3.5 GHz	4.5 GHz	5.25 GHz	5.65 GHz
Predicted with coupling (*f*_2_)	3.85 GHz	5.2 GHz	5.95 GHz	6.25 GHz
Difference (X = *f*_2_ − *f*_1_)	X1 = 0.35 GHz	X2 = 0.7 GHz	X3 = 0.7 GHz	X4 = 0.6 GHz

**Table 3:** Calculated lumped element values.

Circuit	*BW* (MHz)	*Q*_0_	*R* (Ω)	*L* (pH)	*C* (pF)
1	60	59.1	310	239.5	8.65
2	52	88.46	295	127.5	10.3
3	47	111.7	240	67.85	13.54
4	37	156.1	270	47.64	17

**Table 4:** Pulse width stretch ratio and correlation coefficient for the antenna at various distances.

Distance between T_x_ and R_x_ (cm)	Reference Antenna Stretch Ratio (SR)	Proposed Antenna Stretch Ratio (SR)	Reference Antenna Correlation Factor (ρ)	Proposed Antenna Correlation Factor (ρ)
10	1.4	1.95	0.968	0.923
30	1.6	2.20	0.949	0.821
50	1.9	2.37	0.891	0.762

**Table 5:** Comparison between proposed and recently reported UWB antennas in the literature.

Literature	Size (mm)	Filtering Bands (GHz)	Remarks
[23]	28 × 24	N/A	Antenna only operate in UWB range with no rejection bands
[24]	26 × 24	5.1–5.9	Antenna reject the complete WLAN band
[25]	30 × 30	2.4	Antenna rejects 2.4 GHz WLAN band
[26]	40 × 30	3.3–3.7 5.15–5.825	Antenna reject the complete WLAN and WiMAX band, Large size with complicated irregular structure
[27]	45 × 50	5.1–5.825	Rejects complete WLAN band with large dimensions
[28]	22 × 32	4.97–5.28 5.66–5.92	Dismiss the lower and upper WLAN bands, but the notching is not selective
[29]	26 × 21	5.0–6.3	Extra band rejection at the cost of size reduction
[30]	40 × 31	3.31–3.78 5.33–5.77 7.24–7.72	Increased rejection bands at the expense of size. Also, the notching is not selective and reject extra frequency bands
[31]	40 × 20	2.75–3.15 5.15–5.35 7.25–8.39	Inefficient WiMAX and downlink of X-band filtering
[32]	30 × 28	5.15–5.825 3.3–3.76 7.25–7.745	Antenna filtering is not selective at the desired bands and having complex structure
[33]	40 × 20	2.37–2.39 3.27–3.76 5.2–5.89 8.06–8.80	Quad notching without analyzing the coupling b/w CSRR Extra band-notching for WiMAX and X-band satellite communication while complete WLAN band-notching
[34]	33 × 28	3.65 5.75	Compact dimensions with multiple SRR and dual notched bands
[35]	31 × 25	3.4–3.8 5.1–5.35 5.6–6.0 7.15–7.65 8.05–8.65	Compact UWB multiple notched antenna using combination of different slot resonators
[36]	4 × 4.4 cm^2^	5.15–5.825	MEMS-based reconfigurable antenna with fine-tuning of the rejection band
This Work	28 × 30	3.30–3.36 4.50–4.70 5.15–5.35 5.70–5.825	Quad notching with simple structure, compact size, complete and selective filtering with proposed coupling analysis b/w CSRR
